# Sport Dietary Supplements and Physical Activity in Biomedical Students

**DOI:** 10.3390/ijerph18042046

**Published:** 2021-02-19

**Authors:** Dinko Martinovic, Daria Tokic, Marino Vilovic, Doris Rusic, Josipa Bukic, Josko Bozic

**Affiliations:** 1 Department of Pathophysiology, University of Split School of Medicine, 21000 Split, Croatia; d.m.993@hotmail.com (D.M.); marino.vilovic@mefst.hr (M.V.); 2 Department of Anesthesiology and Intensive Care, University Hospital of Split, 21000 Split, Croatia; dariatokic@gmail.com; 3 Department of Pharmacy, University of Split School of Medicine, 21000 Split, Croatia; doris.rusic@mefst.hr (D.R.); josipa.bukic@mefst.hr (J.B.)

**Keywords:** sport dietary supplements, biomedical students, physical activity

## Abstract

Biomedical students should have suitable knowledge about sport dietary supplements (SDS) usage as they are future medical professionals who will have SDS users in their care. The aim of this study was to assess the habits, opinions, and knowledge about SDS usage, along with the level of physical activity, in 386 biomedical students at the University of Split School of Medicine. A specialized questionnaire was developed by a group of experts for the assessment of habits, opinions, and knowledge about SDS and the International Physical Activity Questionnaire—Short Form (IPAQ-SF) was used to evaluate the level of physical activity. The results showed that 49.2% of students used SDS and there was a significant positive correlation between the knowledge questionnaire score and the level of physical activity (r = 0.744, *p* < 0.001). Moreover, SDS users had a higher knowledge questionnaire score (*p* < 0.001) and a higher level of physical activity (*p* < 0.001) compared to non-users. These results suggest that more physically active students are better informed about SDS, but these results also imply that SDS should be implemented in the study program of future medical professionals to ensure that they are informed for their own personal consumption and will be confident in giving advice about SDS usage to their future patients.

## 1. Introduction

As healthy lifestyles have become imperative in modern society, food and supplement consumption habits have also changed. Dietary supplements (DS) are additions to a normal and balanced diet, usually containing a higher level of vitamins, micronutrients, proteins, and other ingredients intended to supplement and balance nutrition [1].

In the last decade, the use of DS has increased among people of all ages. Some of the reasons for this are aggressive marketing, easy access, and a general belief that they reduce the risk of chronic diseases and amplify sports performance with no harmful side-effects for the consumer [2,3,4,5]. Several studies have shown that the most commonly used DS in the general population are vitamins, including vitamin C, vitamin B complex, and multivitamins, followed by minerals such as calcium and zinc [6,7,8,9]. Among more specific populations, the most used DS are folate used during pregnancy and protein supplements used among athletes [10,11]. Another possible reason for the broad usage of DS is that they are mostly self-prescribed and widely available, which gives users free access and lower limitations on consumption [12,13,14].

Sport dietary supplements (SDS) are commonly used among athletes, both professional and amateur, to enhance sports results, achieve better results in less time, and preserve well-being while their bodies undergo a high level of physical effort [15,16,17]. However, SDS are no longer reserved only for the professional athletes, but are also used by the general population, including by individuals who try to maintain a fit lifestyle during their everyday activities and careers. A study by Wardenaar et al. that was conducted on the general population in the Netherlands showed that two thirds of the questioned group used some sort of supplement (DS, SDS, or both), and that men were more likely to use SDS to enhance physical performance and women were more likely to use DS like vitamins for their health benefits [18]. Most DS and SDS are easily accessible and they can be bought in most supermarkets, while basic information about them (both true and false) can easily be found on the internet and forums. Consequently, consumers assume that DS/SDS are not harmful and that they do not need scientific facts or professional advice about DS/SDS usage.

Biomedical students are a specific population with a lack of free time and a great level of stress and anxiety during the period of study. Furthermore, they have access to different types of information, both medical and non-medical, and a commitment to live a healthy lifestyle as an example to the general population [19]. Since biomedical students are future healthcare professionals who will have SDS users in their care, they need to be knowledgeable about the possible adverse effects of SDS usage. Several studies have shown the possible toxicity of inappropriate SDS usage [20,21]. Similar to other medications, SDS also contain physiologically or pharmacologically active substances which could cause adverse effects in vulnerable individuals. Furthermore, certain types of SDS can interfere with some medications, or even be contraindicated for patients with certain diseases [22,23,24].

Several recent studies have shown that biomedical students are better informed about dietary supplementation, its usage, and possible adverse effects compared to other college students [25,26,27]. However, the data regarding the degree of that knowledge in biomedical students and parameters which possibly influence it are still scarce. Hence, the aim of this study was to further investigate the knowledge and opinions about SDS usage among students of medicine, dental medicine, and pharmacy. Moreover, another aim was to evaluate if there is any association between the level of physical activity and knowledge of SDS, and if there are any differences between genders, SDS users, and non-users.

## 2. Materials and Methods

### 2.1. Ethical Considerations and Study Design

This cross-sectional study was conducted at the University of Split School of Medicine during the period from 1 May 2020 to 1 August 2020.

Every subject gave consent to participate by completing and submitting the questionnaire. This research was approved by the Ethics Committee of the University of Split School of Medicine and it was performed according to the latest ethical principles of the Seventh Revision of the Helsinki Declaration from 2013.

### 2.2. Subjects

The survey was conducted on students who were attending study programs of medicine, dental medicine, and pharmacy at the University of Split School of Medicine. Participation in the survey was voluntary and it was performed using a Google Forms^®^ online application which guaranteed the anonymity of provided answers. The survey link was distributed through student organizations and via email. The only exclusion criteria for participation was involvement in professional sport. A total of 798 students were eligible for the survey and we had a response rate of 47%. Thus, 386 students were included in this study.

### 2.3. Physical Activity Questionnaire

Two questionnaires were used in this study. The International Physical Activity Questionnaire—Short Form (IPAQ-SF) is a validated questionnaire which was verified in a Croatian language form [28]. The questionnaire documents the self-reported activity of four intensity levels: vigorous intensity activity such as aerobics; moderate intensity activity such as leisure cycling; walking; and sitting [29,30,31]. The “last 7 days recall” version of the IPAQ-SF for observational studies was used. Metabolic equivalent of task (MET) minutes per week scores were calculated from the results according to the following formulas:

- Walking MET min/week = 3.3 * walking minutes * walking days
- Moderate MET min/week = 4.0 * moderate activity minutes * moderate days
- Vigorous MET min/week = 8.0 * vigorous activity minutes * vigorous days
- Total MET min/week = walking + moderate + vigorous MET min/week scores

The MET values used in the above formulae were derived from a study that validated the reliability of the IPAQ [30].

### 2.4. Sport Dietary Supplementation Questionnaire 

After extensive research of the available literature, we were not able to find any validated questionnaire about the usage of sport dietary supplements suitable for biomedical students, particularly in regard to their knowledge. Hence, a structured, self-administered questionnaire was developed and used for this study. It was created at the Department of Pathophysiology using the most recent and relevant literature. A pilot study was conducted on 41 randomly chosen biomedical students. The average time for the completion of the survey was 10–15 min.

The questionnaire was divided into three sections, with each section consisting of 12 questions. The first section collected socio-demographic data regarding the age, gender, height, body mass, program and year of study, smoking, and history of chronic diseases. The second section collected data regarding the opinions and habits about SDS usage, while the third section was a knowledge questionnaire about SDS usage, benefits, and possible adverse effects.

### 2.5. Development of the SDS Questionnaire 

To ensure the reliability and validity of testing the habits, opinions and knowledge about SDS, a specialized questionnaire was developed by a group of experts. The group consisted of a clinical psychologist, a nutritionist, a pharmacist, and a medical doctor with specialty in sports medicine. They first defined SDS as “any substance which is designed or claimed to improve health and physical condition, enhance athletic performance or help during recovery from an injury”. The questionnaire was divided into two sections: (1) habits and opinions, and (2) knowledge about sport dietary supplements.

In the first section, it was decided that multiple choice questions will be used. According to the characteristics and qualities that were investigated, and with rigorous review of the scientific literature, 14 questions were designed (7 about habits and 7 about opinions). The questions were evaluated by an expert in the Croatian language and revised accordingly. Pre-testing was conducted, and the questionnaire was administered to a sample of 41 randomly chosen biomedical students. Feedback from the respondents showed that all questions were comprehensible and easy to understand. However, 2 questions had a response rate of the answer “Other” >30%, so it was decided to exclude those questions. The final version of the questionnaire consisted of 12 questions (6 about habits and 6 about opinions).

In the second section, two domains in the conceptualization of the questionnaire were distinguished: general knowledge and medical knowledge. It was decided that two types of questions will be used: multiple choice with five possible answers (a, b, c, d, e), and a declarative sentence with a binary response format (true/false). Based on a rigorous review of the scientific and medical literature, 20 questions were designed (10 general knowledge and 10 medical knowledge). The questions were evaluated by a specialist in the Croatian language and revised accordingly. Pre-testing was conducted, and the questionnaire was administered to a sample of 41 randomly chosen biomedical students. The feedback from the respondents showed that the questions were clear and understandable. For each correct answer, one point was assigned and a score was calculated using the sum of all correct answers. Using the results of the pre-testing, we refined the questionnaire by excluding the unreasonably difficult (<10% correct answers) and unreasonably easy (>90% correct answers) questions. The internal consistency of the questionnaire in our sample was acceptable, with a Cronbach’s alpha coefficient of 0.72. The final version of the questionnaire consisted of 12 questions (6 general knowledge, 6 medical knowledge) with a score ranging from 0 to 12.

### 2.6. Statistical Analyses

For the purposes of this study, a free online Surveymonkey^®^ sample size calculator was used. Calculations showed that the minimum sample needed for this study was 260 students according to a 95% confidence interval and a 5% margin of error.

The analysis of the data was performed using MedCalc software for Microsoft Windows (MedCalc Software, Ostend, Belgium, version 17.4.1). Quantitative data are presented as mean ± standard deviation or median and interquartile range, and Student’s t-test or Mann–Whitney U test were used for comparisons between variables. The normality of data distribution was estimated using the Kolmogorov–Smirnov test. Qualitative data are presented as whole numbers and percentages, and the Chi-squared test was used for comparison between variables. Spearman’s rank correlation coefficient was used to test the association between parameters. Furthermore, multiple linear regression analysis was used to determine significant independent predictors of the knowledge questionnaire results. From these analyses, we reported respective *p*-values with unstandardized β-coefficients, standard error, and t-values. Statistical significance was set at *p*-value < 0.05.

## 3. Results

### 3.1. Baseline Characteristics of the Study Population

The study population consisted of 113 (29.3%) male and 273 (70.7%) female students. The mean age was 22.2 ± 1.8 years. Most students were from the medicine program (195; 50%) while there were 119 (30.8%) students from dentistry and 74 (19.2%) from pharmacy (Table 1).

### 3.2. IPAQ Results

In the whole study population, walking MET min/week was 396 (264–594), moderate MET min/week was 240 (120–540), rigorous MET min/week was 360 (0–480), and total MET min/week was 1017 (792–1404) (Table 2).

### 3.3. Habits about SDS Usage

In the whole study population, 190 (49.2%) students answered that they consume SDS regularly. As the first most consumed supplement, 71 (37.4%) students take vitamin C, 61 (32.1%) take whey protein, 12 (6.3%) take magnesium, 21 (11.1%) take a multivitamin, 16 (8.4%) take vitamin B and 9 (4.7%) take other supplements. Most students answered that they buy SDS in pharmacies (101 (53.2%)), while 79 (41.6%) buy them in specialized stores and only 10 (5.3%) on the internet. A minority of students stated that they were recommended to use SDS (112 (29.1%)), most of them by friends (43 (38.4%)), while others were recommended by family (18 (16.1%)), medical professionals (24 (21.4%)), sport coaches (23 (20.5%)) or others (4 (3.6%)). The main source of information about SDS was the internet for 238 (61.7%) students, academic papers for 48 (12.4%) students, medical professionals for 72 (18.6%) students, sport coaches for 19 (4.9%) students, and 9 (2.4%) were not informed (Table 3).

### 3.4. Opinions about SDS Usage

Most students (169 (43.8%)) answered that the main reason for SDS usage among the general population in their opinion is “health quality improvement”, while only 33 (8.5%) students answered “success in sports”. Most of them (279 (72.3%)) believe that SDS are moderately effective, while only 7 (1.8%) think they are ineffective. The main reason for not using SDS was “I don’t need them” from 131 (66.9%) students. For self-reported SDS knowledge grade, only 11 (2.8%) students answered “Excellent”, whereas most (134 (34.7%)) graded their knowledge as “Sufficient” and 76 (19.7%) as “Insufficient”. A minority of students (98 (25.4%)) stated that they recommended SDS to someone, and the main reasons for this were “gaining muscle mass” (28 (28.5%)) and “enhancing athletic performance” (29 (29.5%)) (Table 4).

### 3.5. Knowledge about SDS Usage

**Figure 1:**
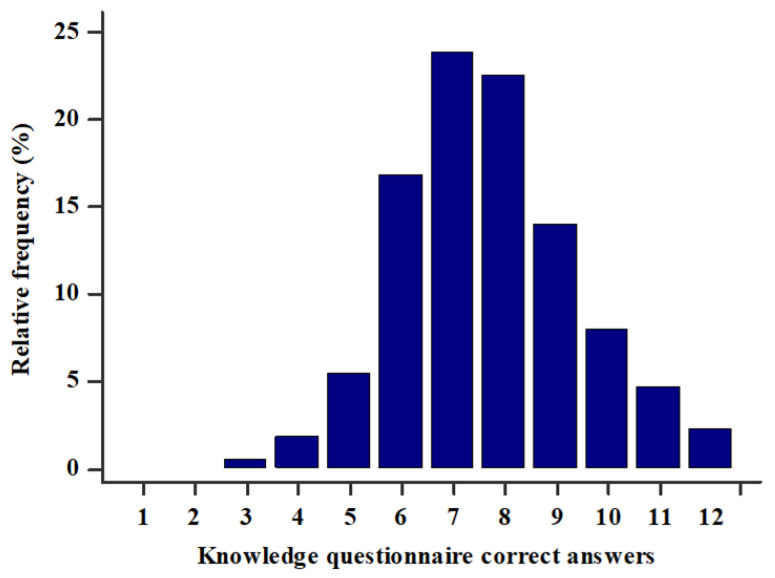
Knowledge questionnaire score (0–12) in the study population (N = 386).

**Figure 2:**
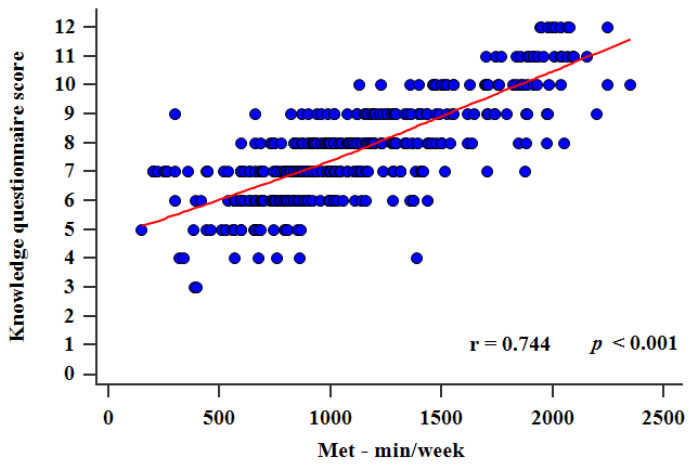
Correlation between total MET scores and knowledge questionnaire score (0–12).

Most students had either 7 (23.8%) or 8 (22.5%) correct answers, and the interquartile range was 7–9 correct answers. Only 9 (2.3%) students correctly answered all 12 questions and only 2 (0.5%) students had the lowest score of 3 correct answers (Figure 1). There was a statistically significant difference between the score in the general knowledge and medical knowledge sections, as students had more correct answers in the general knowledge section (4 (3–5) vs. 4 (3–4), *p* < 0.001). There was a strong positive statistically significant correlation between the knowledge questionnaire score and total MET min/week (r = 0.744, *p* < 0.001) (Figure 2). Furthermore, multiple linear regression analysis showed that the knowledge questionnaire results retained significant association with total MET min/week (*β* ± SE, 0.06 ± 0.02, *p* = 0.003) after model adjustment for study program, study year, gender, and BMI, with the knowledge questionnaire results as a dependent variable (Table 5).

### 3.6. Comparison of SDS User and Non-Users

There was no statistically significant difference between SDS users and non-users regarding age, gender, study program, or study year (*p* > 0.05). However, SDS users had a higher knowledge questionnaire score (8 (7–9) vs. 7 (6–8), *p* < 0.001), higher total MET min/week (1197 ± 468 vs. 1023 ± 444, *p* < 0.001), and higher total Kcal/week during physical activity (1417 ± 737 vs. 1189 ± 668, *p* < 0.001). Moreover, SDS users had significantly more correct answers in the general knowledge section (4 (3–5) vs. 3 (2–4), *p* < 0.001), while there was no significant difference in the medical knowledge section (4 (3–5) vs. 4 (3–5), *p* = 0.942). Furthermore, there was a significant difference in the opinion about the main reason for SDS usage among the general population, as most SDS users answered “health quality improvement” (52.6%), while most non-users answered “physical appearance improvement” (37.8%), *p* < 0.001 (Table 6).

### 3.7. Comparison of Male and Female Students

There was a statistically significant difference between genders, as male students had a higher knowledge questionnaire score (9 (7–10) vs. 7 (6–8), *p* < 0.001), higher total MET min/week (1614 (1212–1885) vs. 1000 (747–1286), *p* < 0.001), and higher total Kcal/week during physical activity (2188 (1710–2656) vs. 932 (710–1167), *p* < 0.001). Moreover, male students had significantly more correct answers in both the general knowledge section (5 (4–5) vs. 4 (3–5), *p* < 0.001) and the medical knowledge section (4 (3–5) vs. 3 (2–4), *p* < 0.001). Furthermore, there was a statistically significant difference in the most consumed supplement, as most males take whey protein (71.7%) and most females take vitamin C (48.5%) (*p* < 0.001). Also, there was a statistically significant difference in the opinion of the main reason for SDS usage among the general population, as most males answered “physical appearance improvement” (50.4%), while females answered “health quality improvement” (54.6%) (*p* < 0.001). Moreover, most males buy SDS in specialized stores (71.7%), while females buy them in pharmacies (68.5%) (*p* < 0.001) (Table 7).

## 4. Discussion

The aim of our study was to investigate knowledge, habits and opinions about SDS usage among biomedical students. Additionally, we wanted to evaluate the possible association between the level of physical activity and knowledge, and to assess if there were any differences between genders, SDS users, and non-users. The results showed that 49.2% of the included students at the University of Split School of Medicine use SDS, and there were no significant differences regarding the study program or the study year. These results are similar to the results of studies conducted on students in the USA (53%) and Australia (56%), while they are lower than those in Serbia (68.1%) [32,33,34]. However, they are significantly higher than the results from similar studies in Portugal (16%), Jordan (27.4%), Korea (31.3%) and even a previous study from Croatia which was conducted in a different county (30.5%) [27,35,36,37]. This can be partly explained by the different trends and opinions about SDS usage in students from different countries and even different regions of the same country. Furthermore, we can explain these differences to some extent by the fact that our study was conducted solely on biomedical students and their level of knowledge about SDS is, as shown by several studies, higher than that of non-biomedical students, which makes them more liable to use SDS [25,26,27].

In the habits section of the questionnaire, students answered that whey protein was the most used supplement (32.1%), followed by vitamin C (37.4%). This is in agreement with previous studies that showed that vitamins are the most used supplements not only among students, but also in the general population, while whey protein is mostly used by athletes, both professional and amateur [5,12,13]. It is interesting that most students (61.7%) stated “internet pages and forums” as their main source of information regarding SDS, while the least of them (5.3%) answered that they buy SDS on the internet. It is possible that they get information about SDS purpose and usage from the internet while they mostly buy them in pharmacies and specialized stores as a guarantee that it is a product of great quality. However, there is a lot of false information, erroneous articles, and incorrect evidence on the internet, and as such it should not be used as the main source of information about SDS, especially by biomedical students who should be taught to use scientific studies, evidence-based articles, and other reliable sources of information. Moreover, most students (43.8%) stated that health quality improvement is the main reason for SDS usage among the general population, while only some (8.5%) see athletic performance as the main reason. Additionally, the main reason for recommending SDS to someone was enhancing their health quality (29.5%). These opinions show that biomedical students see SDS as primarily health beneficial substances, and the possible athletic enhancement is seen as a minor reason for their usage.

In our knowledge questionnaire section, the median score was 8 (7–9) correct answers, which seems to be a relatively low score considering that 6 questions were based on general knowledge and 6 were based on medical knowledge. Moreover, an important finding is that students had more correct answers in the general knowledge section than in the medical knowledge section of the questionnaire. Even though it is expected that biomedical students would be more knowledgeable about the medical aspect, we can explain this finding with the lack of SDS involvement in the curriculum of biomedical studies, and consequently, the possible deficiency in their knowledge regarding the SDS adverse effects, contraindications, and interferences with medications. We found a strong significant positive correlation between the knowledge questionnaire score and the total MET min/week. Moreover, multiple linear regression analysis showed that the knowledge questionnaire score retained significant association with total MET min/week after model adjustment for study program, study year, gender, and BMI with the knowledge questionnaire score as a dependent variable. These results show that the more physically engaged and active students have a better knowledge about SDS. It is possible that their involvement in sport activity and exercise motivates them to investigate and learn more about SDS as a potentially useful tool for better athletic results and recovery after physical activity. This is consistent with recent studies which showed that prior, during, and after their education, biomedical students have the highest motivation to learn and outperform in subjects related to their intrinsic personal interests [38,39,40].

During the comparison of SDS users and non-users, we found that SDS users had a higher score in the knowledge questionnaire and they were more physically active with a higher total MET min/week and consequently a higher Kcal/week during physical activity. These results were anticipated as SDS users are commonly more informed about the substances they are using, and it is well established that the greatest number of SDS users are athletes, both recreational and professional [13,16,17]. However, it is important to highlight the finding that SDS users had more correct answers in the general knowledge section of the knowledge questionnaire, while there was no significant difference in the medical knowledge section. This outcome shows that even though SDS users are more knowledgeable about SDS, it mostly regards general information about SDS usage, while on the other hand, they are still lacking medical knowledge about SDS adverse effects, contraindications, and possible interferences with medications. Another interesting result was the statistically significant difference between SDS users and non-users in the opinion of the main reason for SDS usage among the general population. While most SDS users (52.6%) see the main reason as health quality improvement, the majority of non-users (37.8%) see it as physical appearance improvement. Aesthetics and physical appearance have become a major issue in the last few decades as the image of the perfect male and female bodies are imposed through commercials, movies, magazines, and lately through the internet and social media. Most male and female models that represent these ideal bodies promote SDS as the main means to improve your physical appearance. So, it is possible that underinformed SDS non-users are more likely to link SDS usage and purpose to physical appearance as their main goal of utilization.

Out of the spotlight, but also an interesting result of our study, are the distinctions found in the comparison between male and female students. We found that males have significantly higher knowledge questionnaire scores in both the medical and general knowledge sections. Furthermore, they had significantly higher total MET min/week and consequently a higher Kcal/week during physical activity. Nevertheless, there were no statistically significant differences between male and female students in SDS usage, as both groups were close to a rate of 50% SDS users (*p* = 0.385). These results imply that even though female students are significantly less engaged in physical activity and they have less knowledge about SDS, they are still keen to use SDS at a similar proportion to males. However, most females (38.1%) graded their knowledge about SDS as a 2 (sufficient), while most males (40.7%) graded their knowledge as a 3 (good), which shows that females are self-aware of their lack in knowledge about SDS. Furthermore, the most used SDS by female students is vitamin C (48.5%), while male students mostly use whey protein (71.7%). This significant difference could be associated with the distinctions in the view of the main reason for SDS usage among the general population, as most males answered “physical appearance improvement” (50.4%), while most females answered “health quality improvement” (54.6%). Whey protein is nowadays one of the most used SDS and as a great protein addition, it is mostly associated with muscle mass growth [41,42,43]. Since the imposed image of the perfect male body is muscular, we could reason that there is an association between the view of physical appearance as the main reason for SDS usage and whey protein as the most used SDS in male students. Moreover, male students mostly bought SDS in specialized stores, while females students bought them in pharmacies. Given that vitamin C is not only an SDS, but also a medication for certain conditions, it is possible that female students find pharmacies more reliable as a place for buying it.

The limitations of our study are its cross-sectional design and single center administration. Given that there are a lot of discrepancies between the results of all previous similar studies, a multi-center prospective longitudinal study could provide superior insight about SDS usage among the student population. Furthermore, since we used a questionnaire as the main tool to assess our parameters, there is a possibility that the students overlooked, failed to recall, or had excess subjectivity regarding some of the answers. However, since our study population are future medical professionals who are well aware of ethical principles, we believe in their high moral values and that their answers were honest and reliable.

## 5. Conclusions

In conclusion, the results of our study showed that SDS are frequently used among biomedical students. However, even though we found that SDS users have better knowledge about SDS than non-users, the results of the knowledge questionnaire are not as good as expected from biomedical students, especially in the medical section of the questionnaire. We deem that students should be more informed about the proper management of these substances, their physiological purposes and pathways, possible adverse effects, and contraindications for their usage. Moreover, as most students showed that their main source of information about SDS are internet pages and forums, we consider this a warning for the educational system. SDS usage is on the rise, and as such, SDS information should be incorporated in the curriculum of the future medical professionals to ensure that they are not only informed for their personal consumption, but also for giving advice about SDS usage to their patients.

## Figures and Tables

**Table 1:** Baseline characteristics of the study population.

Parameter	(N = 386)
Male gender (N, %)	113 (29.3)
Age (years)	22.2 ± 1.8
Height (m)	1.74 ± 0.09
Weight (kg)	67.97 ± 13.66
BMI (kg/m^2^)	22.13 ± 2.93
Smoking cigarettes	50 (13)
Medicine students	193 (50)
Dental medicine students	119 (30.8)
Pharmacy students	74 (19.2)
1st year students	47 (12.2)
2nd year students	70 (18.1)
3rd year students	47 (12.2)
4th year students	104 (26.9)
5th year students	58 (15)
6th year students *	60 (15.5)
Family member is a professional sportsman	21 (5.4)
Family member is in health care profession	121 (31.3)
Actively engaged in some sport activity	224 (58)
Hours weekly doing some type of exercise	4 (1–5)

Data are presented as whole numbers (percentages), or mean ± standard deviation, or median (interquartile range (IQR)). * Pharmacy students do not have a sixth year of study. BMI: body mass index.

**Table 2:** International Physical Activity Questionnaire (IPAQ) results.

Parameter	(N = 386)
Walking MET min/week	396 (264–594)
Moderate MET min/week	240 (120–540)
Vigorous MET min/week	360 (0–480)
Total MET min/week	1017 (792–1404)
Kcal/week during physical activity	1092 (808–1709)

Data are presented as median (IQR). MET: metabolic equivalent of task.

**Table 3:** Habits about sport dietary supplement (SDS) usage in the study population.

Parameter	(N = 386)
Using sport dietary supplements	190 (49.2)
Most used supplement	
Whey protein	61 (32.1)
Vitamin C	71 (37.4)
Magnesium	12 (6.3)
Multivitamin	21 (11.1)
Vitamin B	16 (8.4)
Others	9 (4.7)
Where do you buy SDS?	
Specialized store	79 (41.6)
Pharmacy	101 (53.2)
Internet	10 (5.3)
Did someone recommend you SDS?	
Yes	112 (29.1)
No	274 (70.9)
Who gave you the recommendation to use SDS? *	
Friend	43 (38.4)
Family	18 (16.1)
Medical professional	24 (21.4)
Sport coach	23 (20.5)
Other	4 (3.6)
What is your source of information about SDS?	
Internet pages and forums	238 (61.7)
Academic papers	48 (12.4)
Medical professional	72 (18.6)
Sport coach	19 (4.9)
I am not informed	9 (2.4)

Data are presented as whole numbers (percentage). * Only the students who answered Yes to the previous question (N = 112).

**Table 4:** Opinions about SDS usage in the study population.

Parameter	(N = 386)
Main reason for SDS usage among the general population	
Health quality improvement	169 (43.8)
Physical appearance improvement	113 (29.3)
Recovery after injury	59 (15.3)
Success in sports	33 (8.5)
Others	12 (3.1)
Self-reported SDS knowledge grade	
Insufficient	76 (19.7)
Sufficient	134 (34.7)
Good	126 (32.6)
Very good	39 (10.1)
Excellent	11 (2.8)
How effective are SDS in your opinion?	
Very effective	14 (3.6)
Moderately effective	279 (72.3)
Somewhat effective	86 (22.3)
Ineffective	7 (1.8)
If you do not use SDS, what is the reason?	
They are too expensive	13 (6.6)
They are harmful	7 (3.6)
I do not need them	131 (66.9)
I do not know enough about them	45 (22.9)
Did you recommend someone to use SDS?	
Yes	98 (25.4)
No	288 (74.6)
What was the reason to recommend SDS to someone? *	
Losing weight	8 (8.1)
Muscle soreness	11 (11.2)
Fatigue	7 (7.1)
Enhancing immunity	28 (28.5)
Enhancing athletic performance	29 (29.5)
Other	15 (15.3)

Data are presented as whole numbers (percentage). * Only the students who answered Yes to the previous question (N = 98).

**Table 5:** Multiple linear regression model of independent predictors for knowledge questionnaire results.

Variable	*β* *	SE ^†^	*t* Value	*p*
Study program	−0.025	0.076	−0.335	0.737
Study year	0.014	0.036	0.410	0.681
Gender	0.168	0.172	0.974	0.330
BMI	−0.015	0.022	−0.675	0.500
Total MET (min/week)	0.002	0.0001	19.246	<0.001

* unstandardized coefficient β. ^†^ standard error.

**Table 6:** Comparison between SDS users and non-users.

Parameter	SDS Users (N = 190)	SDS Non-Users (N = 196)	*p* *
Male gender	60 (31.6)	53 (27.0)	0.385
Age (years)	22.35 ± 1.73	22.13 ± 1.92	0.239
BMI (kg/m^2^)	22.26 ± 2.81	21.99 ± 3.04	0.363
Knowledge questionnaire score	8 (8–9)	7 (6–8)	<0.001
General knowledge section score	4 (3–5)	3 (2–4)	<0.001
Medical knowledge section score	4 (3–5)	4 (3–5)	0.942
Total MET (min/week)	1197 ± 468	1023 ± 444	<0.001
Total Kcal/week	1417 ± 737	1189 ± 668	<0.001
Study program			0.613
Medicine	99 (52.1)	94 (48.0)	
Dental medicine	58 (30.5)	61 (31.1)	
Pharmacy	33 (17.4)	41 (20.9)	
Study year			0.502
1st year students	22 (11.6)	25 (12.8)	
2nd year students	30 (15.8)	40 (20.4)	
3rd year students	24 (12.6)	23 (11.7)	
4th year students	51 (26.8)	53 (27.0)	
5th year students	35 (18.4)	23 (11.7)	
6th year students ^†^	28 (14.7)	32 (16.3)	
Main reason for SDS usage among the general population			<0.001
Health quality improvement	100 (52.6)	69 (35.2)	
Physical appearance improvement	39 (20.5)	74 (37.8)	
Recovery after injury	40 (21.1)	19 (9.7)	
Success in sports	7 (3.7)	26 (13.3)	
Others	4 (2.1)	8 (4.0)	

Data are presented as whole numbers (percentage) or mean ± standard deviation or median (IQR). * *t* test for independent samples, Mann–Whitney U test or chi-square test. ^†^ Pharmacy students do not have the sixth year of study.

**Table 7:** Comparison of male and female students.

Parameter	Males (N = 113)	Females (N = 273)	*p* *
Age (years)	22.31 ± 1.88	22.21 ± 1.81	0.618
BMI (kg/m^2^)	24.49 (23.05–25.64)	20.72 (19.47–22.28)	<0.001
Using dietary supplements	60 (53.1)	130 (47.6)	0.385
Knowledge test score	9 (7–10)	7 (6–8)	<0.001
General knowledge section score	5 (4–5)	4 (3–5)	<0.001
Medical knowledge section score	4 (3–5)	3 (2–4)	<0.001
Total MET (min/week)	1614 (1212–1885)	1000 (747–1286)	<0.001
Kcal/week during physical activity	2188 (1710–2656)	932 (710–1167)	<0.001
Most used supplement			<0.001
Whey protein	43 (71.7)	18 (13.8)	
Vitamin C	8 (13.3)	63 (48.5)	
Magnesium	1 (1.7)	11 (8.5)	
Multivitamins	3 (5.0)	18 (13.8)	
Vitamin B	3 (5.0)	13 (10.0)	
Others	2 (3.3)	7 (5.4)	
Self-reported SDS knowledge grade			<0.001
1—Insufficient	11 (9.7)	65 (23.8)	
2—Sufficient	30 (26.5)	104 (38.1)	
3—Good	46 (40.7)	80 (29.3)	
4—Very good	22 (19.5)	17 (6.2)	
5—Excellent	4 (3.5)	7 (2.6)	
Main reason for SDS usage among the general population			<0.001
Health quality improvement	20 (17.7)	149 (54.6)	
Physical appearance improvement	57 (50.4)	56 (20.5)	
Recovery after injury	22 (19.5)	37 (13.6)	
Success in sports	9 (8.0)	24 (8.8)	
Others	5 (4.4)	7 (2.5)	
Where do you buy SDS			<0.001
Specialized store	43 (71.7)	36 (27.7)	
Pharmacy	12 (20.0)	89 (68.5)	
Internet	5 (8.3)	5 (3.8)	

Data are presented as whole numbers (percentage), mean ± standard deviation and median (IQR). * *t* test for independent samples, Mann–Whitney U test or chi-square test.

## Data Availability

All data is available from the corresponding author.
